# Temporary percutaneous trans-tibiofibular K-wire stabilization of selected unstable ankle fractures

**DOI:** 10.2340/17453674.2026.46960

**Published:** 2026-10-28

**Authors:** Anders F OVERGAARD, Thomas BLOCH, Chris H DREYER

**Affiliations:** Department of Orthopaedic Surgery and Traumatology, CORI – Centre for Orthopaedic Research and Innovation, Central and West Zealand Hospital, Denmark

Temporary stabilization of an unstable ankle fracture is required when closed reduction cannot be maintained and immediate open reduction and internal fixation (ORIF) for some reason is unsuitable or needs to be postponed. We describe a minimally invasive trans-tibiofibular technique using a single 2.8-mm threaded K-wire. Instability was defined as persistent talar malalignment following closed reduction or recurrent displacement requiring operative stabilization, which will usually be performed by bilateral delta frame. The construct permits circumferential compression, soft-tissue inspection, and use of a removable walker boot. This technical report provides a reproducible stepwise method for selected fracture patterns.

## Patient selection and operative preparation

The proposed indication is an adult with a closed unstable AO/OTA 44A–C ankle fracture, persistent talar malalignment, or recurrent displacement after closed reduction and immobilization, and a clinical need for temporary fixation before ORIF. Immediate ORIF remains preferable when feasible. Splinting and frame fixation are established interim options [1,2]. A percutaneous wire should be used only if reduction can be restored and the fracture configuration permits adequate stability with this construct. Persistent instability requires another fixation strategy.

Open fractures, pilon fractures, posterior fracture-dislocations, complex articular injuries, and polytrauma were outside the intended indication for this technique. These injuries may require debridement, additional stabilization, or a different treatment sequence; the exclusions do not imply that a wire could never be useful in an individual case. Active infection, severe peripheral vascular disease, and a wire trajectory that cannot avoid the joint are further reasons to choose another approach.

Place the patient supine on a radiolucent table, with the limb supported and the foot in neutral alignment. Prepare and drape the leg, administer antibiotic prophylaxis according to local practice, and use fluoroscopy throughout. A right-handed surgeon may stand to the patient’s left and use the non-dominant hand to maintain reduction and guide the trajectory (Figure 1). Required instruments include a scalpel, blunt dissector, 3.2-mm drill, and 2.8-mm threaded K-wire (Arthrex, Naples, FL, USA).

**Figure 1:**
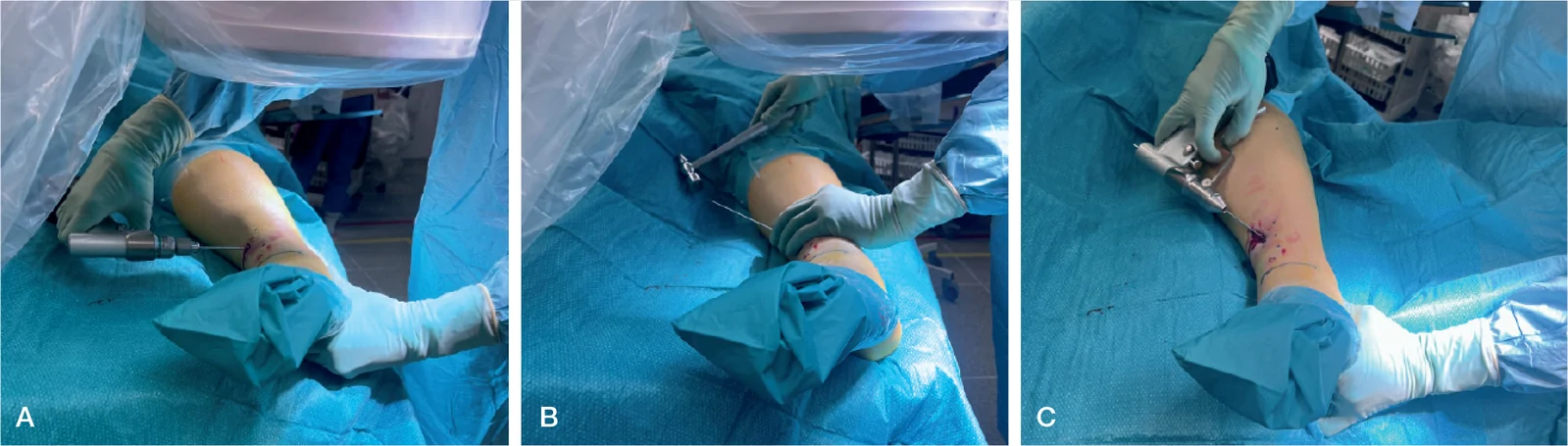
Surgeon position and fluoroscopic guidance: (A) maintenance of reduction and planned trajectory; (B) preliminary wire alignment; (C) final wire advancement.

## Wire trajectory and fixation

Obtain an AP-mortise view after closed reduction. Plan a line toward the distal fibular tip, crossing the lateral clear space about 5 mm proximal to the joint line; the medial tibial entry is approximately 6–7 cm proximal to the plafond (Figure 2). Confirm that this trajectory remains extra-articular in both AP and lateral views.

**Figure 2:**
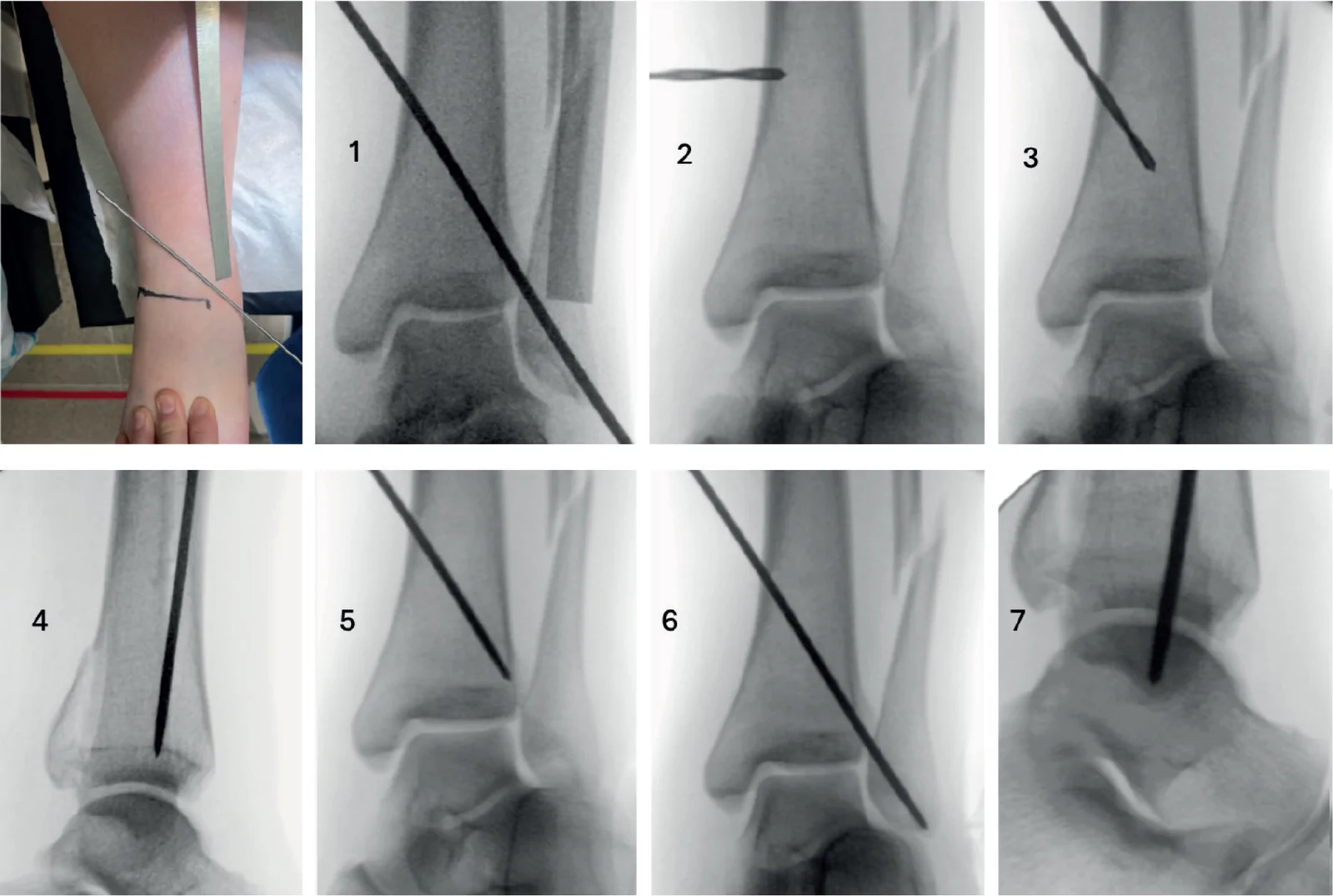
Planning and key steps of the procedure, with final AP and lateral fluoroscopic images showing trans-tibiofibular wire placement and ankle-mortise alignment.

## Cortical entry

Make a short incision approximately 1 cm medial to the anterior tibial crest and dissect bluntly to bone. Drill the near tibial cortex initially perpendicular to the shaft, then angle the channel toward the distal fibular tip. Do not advance the drill beyond the central tibial cancellous bone (Figure 2).

## Wire insertion

Introduce the threaded wire through the prepared channel and advance it to, but not through, the far tibial cortex. Recheck ankle reduction and the planned course on AP and lateral fluoroscopy. If satisfactory, advance the wire through the far tibial cortex and into the distal fibula, ending near its tip without entering the ankle joint (Figures 1 and 2). Avoid repeated passes when crossing the fibula.

## Final assessment and interval care

Confirm mortise congruency, wire position, and absence of joint penetration on AP, mortise, and lateral views. Assess stability; if reduction cannot be maintained, use an alternative method of temporary fixation. Cut the wire above skin level, apply a sterile pin-site dressing and circumferential compression, and protect the limb in a removable walker boot as appropriate. Reassess skin, pin site, neurovascular status, and alignment during the interval to ORIF. Remove the wire at definitive fixation and manage the entry site according to local protocol.

## Perspective

This report describes a reproducible, minimally invasive option for temporary stabilization when closed reduction cannot be maintained and definitive ORIF must be delayed. By stabilizing the distal fibula to the tibia, the construct is intended to support fibular length and talar alignment, which are central to ankle-mortise congruency [1,3]. The percutaneous approach also leaves the ankle accessible for compression and repeated soft-tissue assessment. Compression can reduce post-traumatic edema [4], and this may be relevant when definitive fixation is deferred, as delay has been associated with increased wound complications [2,5].

For lateral malleolar fractures, recent guidance highlights the role of weightbearing radiographs in assessing mortise congruency when deciding whether surgery is needed [6]. This reinforces the need to establish true instability rather than infer it from the fracture label alone. The technique described here is intended for the broader group of ankle fractures judged to require surgery, when reduction cannot be maintained and definitive ORIF is delayed.

The technique should not be considered a replacement for prompt ORIF when feasible or for external fixation when the injury pattern or soft tissues require a frame. Open fractures, severe instability, complex articular injuries, and unsuitable soft-tissue conditions may require another strategy [7]. Although the K-wire may provide a practical means of maintaining talar alignment in selected cases, this technical report does not establish its safety, complication rate, or superiority over established methods. Prospective evaluation should first assess safety and feasibility, followed by comparative studies of reduction maintenance, soft-tissue complications, infection, patient-reported outcomes, resource use, and time to definitive surgery.

## Notes

Handling co-editor: Cecilia Rogmark

*Acta* thanks Emilia Möller Rydberg and Daniel Wenger for help with peer review of this manuscript.
